# Up to the TASG: a participatory study on sexual health of trans and non-binary persons in Germany

**DOI:** 10.1093/eurpub/ckac131.506

**Published:** 2022-10-25

**Authors:** U Koppe, C Spurgat, JM knoop, A Hahne, MN Appenroth, K Pöge, J Hamm

**Affiliations:** 1 Department of Infectious Disease Epidemiology, Robert Koch Institute, Berlin, Germany; 2 Deutsche Aidshilfe, Berlin, Germany; 3 Kiel, Germany; 4 Hamburg, Germany; 5 Institute of Public Health, Charité Universitätsmedizin Berlin, Berlin, Germany

## Abstract

**Problem:**

Comprehensive data on the sexual health of trans and non-binary people are not available due to lacking focus on these groups and inappropriate study designs that often fail to capture the lived realities of these communities.

**Description of the practice:**

A participatory study was developed with trans and non-binary representatives with a qualitative part involving single and group interviews as well as the development and roll-out of a quantitative online questionnaire. After securing funding, we started the study in 2020 ensuring community involvement in as many parts as possible.

**Results:**

A crucial component is the advisory board including people representing a broad spectrum of trans and non-binary communities, organizations, who are also representing a variety of intersectional perspectives, e.g. BIPoC and neurodiverse people. The advisory board provides crucial input to the design and conduct of the study components. For the qualitative part, a unique study design was developed using sexual health & empowerment workshops for trans and non-binary people in a trustful peer setting combined with qualitative data collection. This way, participants of the study truly benefit from participation and the data quality is improved. The quantitative study was created together with >20 community representatives. Items on gender identity, transition, and experiences of discrimination were developed using online workshops and written feedback. The questionnaire was checked by community members to ensure appropriate language. Through diverse community channels and social media, we recruited 58 participants for the qualitative part and more than 2000 participants for the online questionnaire. The results are expected in late 2022.

**Lessons:**

Involvement of community representatives and the participatory study design ensured strong community support. This enables the capture of meaningful information on the sexual health of trans and non-binary people.

**Key messages:**

• Using a participatory study design was crucial to the success of this study.

• We were able to conduct a study capturing meaningful data on the sexual health of trans and non-binary communities.

